# The Gene Encoding Subunit A of the Vacuolar H^+^-ATPase From Cotton Plays an Important Role in Conferring Tolerance to Water Deficit

**DOI:** 10.3389/fpls.2018.00758

**Published:** 2018-06-07

**Authors:** Na Liu, Zhiyong Ni, Haiyan Zhang, Quanjia Chen, Wenwei Gao, Yongsheng Cai, Mengyu Li, Guoqing Sun, Yan-ying Qu

**Affiliations:** ^1^College of Agronomy, Xinjiang Agricultural University, Ürümqi, China; ^2^Biotechnology Research Institute, Chinese Academy of Agricultural Sciences, Beijing, China

**Keywords:** cotton (*Gossypium hirsutum*), vacuolar H^+^-ATPase gene, gene expression, water deficit tolerance, virus-induced gene silencing

## Abstract

In plant cells, vacuolar H^+^-ATPases (V-ATPases) are responsible for deacidification of the cytosol and energisation of the secondary transport processes across the tonoplast. A number of V-ATPase subunit genes have been demonstrated to be involved in the regulation of the plant response to water deficit. However, there are no reports on the role of V-ATPase subunit A (VHA-A) in dehydration tolerance of cotton. In this study, cotton *GhVHA-A* gene was functionally characterized, especially with regard to its role in dehydration stress tolerance. Expression analysis showed that *GhVHA-A* was differentially expressed in various cotton organs and was induced by dehydration, low temperature, high salinity, and abscisic acid treatment in leaves. We also report that *GhVHA-A* improve dehydration tolerance in transgenic tobacco and cotton. Virus-induced gene silencing of *GhVHA-A* decreased the tolerance of cotton plantlets to dehydration stress. Silencing *GhVHA-A* decreased chlorophyll content and antioxidant enzyme activities and increased malondialdehyde (MDA) content in cotton under dehydration stress. However, transgenic tobacco expressing *GhVHA-A* exhibited enhanced dehydration resistance, resulting in reduced leaf water loss, higher average root length, and lower MDA levels under dehydration stress. Meanwhile, overexpression of *GhVHA-A* in tobacco conferred water deficit tolerance by enhancing osmotic adjustment (proline) and the activities of the antioxidant enzymes superoxide dismutase and peroxidase, thereby enhancing reactive oxygen species detoxification. These results suggest that *GhVHA-A* plays an important role in conferring resistance to dehydration stress. Our results have identified *GhVHA-A* as a candidate gene for improving dehydration tolerance in plants.

## Introduction

One way to improve plant drought tolerance is to increase the activity of the H^+^ pump on the tonoplast, allowing more H^+^ to enter the vacuole, producing a higher proton electrochemical gradient (H^+^) and increasing the solute concentration in plant cell vacuoles (Osmotic pressure regulation) and vacuolar osmotic pressure. This decreases water potential, favoring moisture transport from the soil into plant root cells. The electrochemical H^+^ gradient is produced by two H^+^-pumps: that is, vacuolar H^+^-inorganic pyrophosphatase (V-PPase; EC 3.6.1.1) and vacuolar H^+^-ATPase (V-ATPase; EC 3.6.1.3) in plants. V-PPase is a unique proton pump that composed of a single polypeptide, as a dimer of 71–80 kDa subunits, and has only been identified in plants and some algae, bacteria, protozoa, and archaebacterial (Maeshima, 2000). At this time, the V-PPase has been well characterized, and the heterologous overexpression of analogous genes encoding vacuolar membrane-bound pyrophosphatase (H^+^ PPase or H^+^ pump) from rice, tobacco, cotton and maize enhances salt and drought tolerance (Gao et al., 2006; Zhao et al., 2006; Li et al., 2008; Lv et al., 2008).

However, the study of V-ATPase is difficult since it consists of many subunits. In plants, the vacuolar H^+^-ATPase constitutes a large multimeric enzyme complex that transports protons across the membrane through primary active transport. V-ATPase is important for the maintenance of homeostasis in eukaryotic cells (Gluck, 1993; Harvey and Wieczorek, 1997). V-ATPase forms multi-subunit enzyme complexes with a molecular weight of 450∼600 kDa localized to vacuoles, and conserved in all eukaryotes (Sze et al., 2002). The V-ATPase is comprised of two domains: a large cytosolic V1 domain, which has eight subunits (A through H), and a membrane-bound proton-translocating V0 domain, comprising subunits a, c, c′, c″, d, and e. The V-ATPase A subunit (i.e., VHA-A), responsible for ATP hydrolysis, is one of the most highly conserved eukaryotic proteins (Gaxiola et al., 2007). In most organisms, subunit A has a molecular weight of 70 kDa and is a hydrophilic peptide located in the head group at the peripheral portion of V1, with each holoenzyme consisting of three copies. Subunit A also contains a highly conserved cysteine residue that may be involved in the regulation of holoenzyme, which is located in the enzyme catalytic center (Forgac, 1989).

Previous studies have shown that genetic modification technology has enhanced the abiotic tolerance of plants. Overexpression of the *V-PPase* gene can improve drought and salt tolerance in many transgenic plants, including *Arabidopsis* (Gaxiola et al., 2001; Dong et al., 2011; Pasapula et al., 2011). Although the structure of the V-ATPase is complex, there have been reports showing that it can improve the tolerance of plants under abiotic stress. For example, *SaVHAc1* (Baisakh et al., 2012), *IrlVHA-c* (Wang et al., 2016), and *OsVHA-A* (Zhang et al., 2013) play important roles in modulating tolerance to drought and salinity. Moreover, over-expression of *VHP* has been shown to enhance drought resistance of cotton (Lv et al., 2009). To date, however, there are no reports on the role of VHA-A in dehydration tolerance of cotton.

In a previous study exploring the effects of dehydration stress on cotton seedlings, 110 protein spots were detected on two-dimensional polyacrylamide gel electrophoresis (2-DE) maps, 56 of which were identified by matrix-assisted laser desorption ionization time-of-flight (MALDI-TOF) and MALDI-TOF/TOF mass spectrometry. The V-ATPase was significantly upregulated at 24 h in response to dehydration stress (Zhang et al., 2016). We cloned and characterized the complementary DNA (cDNA) of the *GhVHA-A* gene in *Gossypium hirsutum* in a previous study (Liu et al., 2015). In this report, we characterized the expression pattern and examined the involvement of the H^+^-ATPase subunit A (*GhVHA-A*) gene in the response to dehydration stress through a virus-induced gene silencing (VIGS) approach. To further characterize *GhVHA-A*, we generated transgenic tobacco plants constitutively overexpressing *GhVHA-A*, and found that transgenic plants had enhanced dehydration stress tolerance. This study sought to elucidate the role of *GhVHA-A* in dehydration stress responses.

## Materials and Methods

### Plant Materials, Growth Conditions, and Stress Treatments

Cotton cultivar KK1543 (*G. hirsutum*) was grown as previously described (Zhang et al., 2016). For dehydration, salinity, and abscisic acid (ABA) treatments, at the three-leaf stage, some of the seedlings were treated with 1/2 Hoagland’s nutrient solution containing 15% polyethylene glycol (PEG), 250 mM NaCl, and 100-μM ABA, respectively. Other seedlings were incubated at 4°C for the low-temperature treatment. The remaining seedlings were transferred to normal nutrient solution and served as controls. Leaves of treated and control seedlings were collected separately at 2, 4, 6, 12, and 24 h after treatment, frozen immediately in liquid nitrogen, and stored at -80°C for RNA preparation. In addition, the roots, stems, and leaves under normal conditions were sampled for RNA isolation and organ-specific expression analysis. Each treatment was performed in three replicates of 10 plants each.

Cotton cultivars (Xinluzao 26, Xinpao 1, Kui 85174, ND359-2, Xinluzao 36, Shiyuan 321, KK1543, Zhong R773-1, Xinluzhong 3, Xinluzao 38, Xinluzao 35, and C6015) with different drought sensitivities were used (Liu et al., 2014, 2016). The drought coefficient of cotton cultivars was obtained as described previously (Liu et al., 2016). Cotton seedlings were treated with 15% PEG for 24 h. Leaves were harvested, frozen immediately in liquid nitrogen, and stored at -80°C until RNA preparation. All cotton seedlings were grown in a growth chamber under controlled conditions at 28°C and a 16-h light/8-h dark photoperiod.

Tobacco plants (*Nicotiana tabacum* NC89) were used for gene transformation. Wild-type (WT) tobacco seeds were germinated in Petri dishes containing Murashige-Skoog (MS) medium with 0.6% (w/v) agar. Homozygous lines selected from T_2_-generation seeds (see the section on transformation for details) were sown in pots containing a soil mix (1:1.5, vermiculite:humus). Tobacco plants were incubated at 25°C with a 16-h light/8-h dark photoperiod.

### Quantitative Real-Time Polymerase Chain Reaction (qPCR)

Total RNA was extracted using a Total RNA Plant Extraction kit (Tiangen, Beijing, China) according to the manufacturer’s instructions. First-strand cDNA was synthesized using a First-Strand cDNA Synthesis kit (Thermo, Shanghai, China) according to the manufacturer’s protocol. For qRT-PCR, primers specific for *GhVHA-A* were designed (forward primer 5′-GACTCTGCTACAATCCAAGTTTATGAAGA-3′ and reverse primer 5′-TAGTTTTCAAAGGCCTCTGAATGCC-3′). The *UBQ7* gene (amplified by forward primer 5′-GACCTACACCAAGCCCAAGAAG-3′ and reverse primer 5′-TGAGCCCACACTTACCACAATAGT-3′) was used as an endogenous control. *N. tabacum* genes (18S ribosomal RNA) were used as internal references in the qRT-PCR the analysis of transcript levels of *GhVHA-A* in transgenic tobacco. qRT-PCR analysis of genes was performed using the TransStart Tip Green qPCR SuperMix (TransGen Biotech, Beijing, China) on an ABI 7500 Real-Time PCR System (Applied Biosystems, Foster City, CA, United States). Gene expression was quantified using the comparative Ct: 2^-ΔΔCt^ method (Livak and Schmittgen, 2001). Means of three biological replicates and standard deviations were calculated.

### VIGS and Gene Expression Analysis in Cotton

*Tobacco rattle virus* (*TRV*) was previously suggested to be effective in silencing endogenous genes in cotton through VIGS (Gao et al., 2011; Mustafa et al., 2016). In this study, a 340 base pair fragment was amplified from the *GhVHA-A* gene via PCR using primers 5′-AATGAATTCGGACGGTCAGAAGATAACATA-3′ and 5′-TTTGGTACCGCCAGAAAG TAATAAATAACAAAT-3′. The PCR product was inserted into plasmid pTRV2 to produce pTRV2-*GhVHA-A*. Recombinant plasmid pTRV2-*GhVHA-A* was introduced into the *Agrobacterium tumefaciens* strain GV3101. The agroinfiltration method used in cotton for VIGS was performed as described previously. The cotton *CLA1* gene was used as a positive control to supervise VIGS efficiency (Gao et al., 2011). VIGS experiments were repeated at least three times with more than eight plants for each construct per repeat. Total cotton RNA was extracted from leaves of VIGS plants at approximately 2 weeks after VIGS using a Total RNA Plant Extraction Kit (Tiangen) according to the manufacturer’s instructions. qRT-PCR was used to analyze the *GhVHA-A* expression in VIGS plants.

### Dehydration Treatments in Cotton

Two different experiments, water-withholding treatment and PEG treatment, were performed to explore the function of *GhVHA-A* in the cotton response to water deficit. Prior to dehydration treatment, all cotton plants were regularly irrigated for 7 days. In the PEG treatment experiment, *GhVHA-A*-silenced (i.e., VIGS), empty vector pTRV2-treated (i.e., EM), and WT seedlings at the three-leaf stage were treated with 200 mL of 15% PEG 6000 solution and incubated for 24 h. For the water-withholding treatment, VIGS, EM, and WT plantlets at the three-leaf stage were deprived of water for 15 days, after which the extent of cotton withering was observed.

### Transformation and Identification of Transgenic Tobacco Plants

The coding region of *GhVHA-A* was amplified using specific primers (forward primer 5′-TTTCCATGGATGCCGGCAGTTTACGGATCCAGA-3′ and reverse primer 5′-AAAACTAGTCCTAGTTTCATCCTCCAATGCACG-3′) containing *Spe* I and *Nco* I restriction enzyme sites. The coding region was inserted into the vector pCAMBIA1304 downstream of the CaMV35S promoter. The resultant vector was introduced into *Agrobacterium tumefaciens* EHA105 and transformed into *N. tabacum* (NC89) ecotype tobacco plants using the leaf disk transformation method (Horsch et al., 1985).

DNA was extracted from WT and T_0_ hygromycin-resistant plants and subjected to PCR using a pCAMBIA1304-specific forward primer (5′-GAGAACACGGGGGACTCTTGA-3′) and *GhVHA-A*-specific reverse primer (5′-AAAACTAGTCCTAGTTTCATCCTCCAATGCACG-3′). This yielded two independent transgenic *GhVHA-A*-overexpressing *N. tabacum* lines, named V-3 and V-7. Stable expression of the transgene in the progeny was verified by qRT-PCR. Homozygous lines selected from T_2_ generations were used in subsequent experiments.

### Dehydration Treatment of Transgenic Tobacco

For dehydration tolerance assays, transgenic and WT seeds were sown in pots containing a soil mix (1:1.5, vermiculite:humus). For dehydration treatment, 4-week-old transgenic and WT tobacco plants were grown in pots and adequately watered. Subsequently, water was withheld for approximately 15 days, and the survival rates were recorded after re-watering for 7 days. Dehydration tolerance experiments were conducted in triplicate. The numerical data were subjected to statistical analyses using Excel 2003 (Microsoft, Redmond, WA, United States).

### Water Loss Measurement

The water loss assay was performed as described by Chen et al. (2015). Each treatment was performed in three replicates of 10 plants each.

### Leaf Disks Treated With PEG 6000

Leaf disks (8-mm diameter) from the youngest, fully expanded leaves were cut using a cork borer and soaked in 20% PEG 6000 solution for 1 week. WT tobacco leaf disks treated with distilled water served as controls. All leaf disks were incubated at 25°C with a 16-h light/8-h dark photoperiod. Each treatment was performed in three replicates of 20 plants each.

### Root Length Assay

For the root length assay, 2-week-old transgenic and WT seedlings were deprived of water for 7 days, followed by re-watering once. Root lengths were measured after 3 days of growth. At the same time, the root lengths of WT and transgenic plants under normal growth conditions were measured. Each treatment was performed in three replicates of 10 plants each.

### Determination of Malondialdehyde (MDA) and Proline Content and Reactive Oxygen Species (ROS) Staining

Malondialdehyde content analysis as described by Sekmen et al. (2014). Determination of proline content using the acid-ninhydrin method (Bates et al., 1973). Accumulation of H_2_O_2_ and O_2_^-^ in dehydrated leaves was investigated *in situ* by histochemical staining with 3,3′-diaminobenzidine (DAB) and nitroblue tetrazolium (NBT), respectively, according to Wang et al. (2011). In brief, for localization of H_2_O_2_, leaves were placed in 1 mg ml^-1^ DAB solution (pH 3.8) for 24 h at 25°C in the dark. For O_2_^-^ detection, leaves were incubated in 1 mg ml^-1^ NBT solution prepared in 10 mM phosphate buffer (pH 7.8) at 25°C under light until blue spots appeared. The stained leaves were immersed in 75% ethanol to remove chlorophyll. Each treatment was performed in three replicates of 20 leaves each.

### Measurement of Chlorophyll Content and Relative Conductivity

The relative conductivity of the leaves was measured using the suction method, as described previously (Institute of Plant Physiology and Ecology et al., 1999). Chlorophyll extracted from 0.5 g leaf tissue in ice-cold acetone: absolute ethyl alcohol mix (1:1 volume/volume) was centrifuged and the supernatant was made up to a known volume (10 mL). Absorbance was recorded at 645 and 663 nm using a UV-visible spectrophotometer (T6, Persee Corporation, Beijing, China). Total chlorophyll content per gram FW of leaf was estimated as described by Lichtenthaler (Hiscox and Israelstam, 1979). Each assay was performed at least in triplicate for each sample.

### Antioxidant Enzyme Activity Assay

The activities of the antioxidant enzymes superoxide dismutase (SOD; EC 1.12.1.11) and peroxidase (POD; EC 1.11.1.7) in leaves were estimated, as described previously (Li et al., 2010). In brief, the activity of SOD was measured at A_560_, in a 3 ml reaction mixture (13 mM _DL_-methionine, 10 μM EDTA, 75 μM Nitro Blue tetrazolium chloride and 2 μM riboflavin in 50 mM phosphate buffered saline, pH 7.8, and 50 μL protein extract). The activity of POD was measured at A_470_, in a 5 ml reaction mixture (2% H_2_O_2_ and 50 mM guaiacol in 50 mM phosphate buffered, pH 5.5, and 500 μL protein extract). Total antioxidant capacity (T-AOC) was determined by measuring antioxidant proteins using a colorimetric assay (Nanjing Jiancheng Bioengineering Institute, Nanjing, China). Each assay was performed at least in triplicate for each sample.

### Expression Analysis of the Antioxidant Enzyme-Coding and Stress-Related Genes

Four-week-old tobacco plants in soil were withheld watering for 7 days. The leaves of dehydration-treated plants were detached and subjected to RNA extraction, and the RNA samples were used to analyze the expression of antioxidant enzyme-coding genes (*SOD*, AB093097; *POD*, ACN91229.1). In addition, samples were used for expression analyses of the stress-responsive genes *NtLEA5*, *NtERD10D*, and *NtNCED3-1*.

### Statistical Analysis

The data were subjected to Student’s *t*-test analysis using SPSS statistical software 18.0 (SPSS Inc., United States).

## Results

### Expression Profiles of *GhVHA-A* in Cotton

**FIGURE 1:**
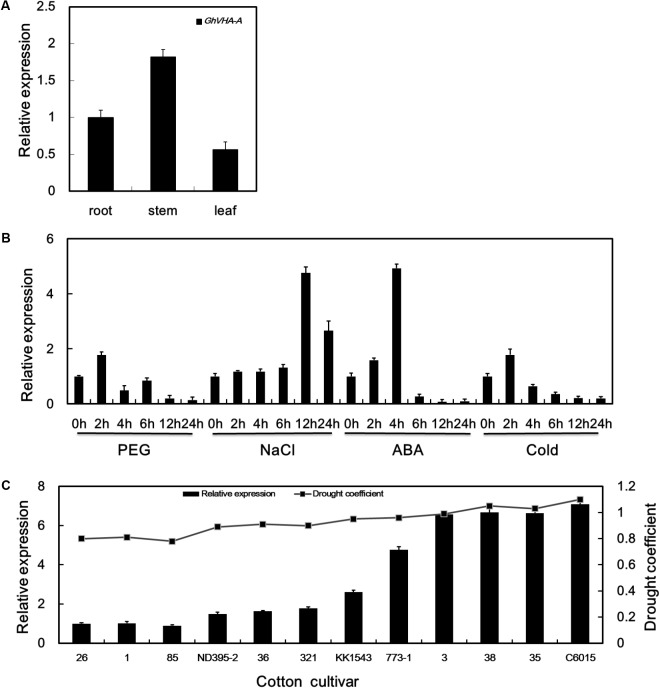
Expression pattern of vacuolar H^+^-ATPase (V-ATPase). **(A)** Relative expression of *GhVHA-A* in various organs. **(B)** Expression profiles of *GhVHA-A* under abiotic stress conditions. **(C)** *GhVHA-A* expression in cotton cultivars under dehydration stress. Values represent the means of three biological replicates, and error bars represent standard deviations.

Under dehydration stress conditions, the V-ATPase catalytic subunit was upregulated during the seedling-stage of KK1543 drought-tolerant cotton at 24 h (**Supplementary Figure S1**), as determined by 2-DE and MALDI-TOF mass spectrometry (Zhang et al., 2016). Full-length cDNA of *GhVHA-A* was obtained and characterized as described previously (Liu et al., 2015). qRT-PCR was further used to analyze the *GhVHA-A* expression pattern. As shown in **Figure 1A**, *GhVHA-A* was constitutively expressed in roots, stems, and leaves. Expression was higher in the stems than in the other organs examined. Using qPCR, *GhVHA-A* was found to be upregulated by dehydration, salt, ABA, and low-temperature treatments (**Figure 1B**). During dehydration treatment, *GhVHA-A* expression peaked (1.7-fold induction) at 2 h, and then decreased thereafter. Upon application of salt stress, the expression of *GhVHA-A* increased gradually, peaked (4.7-fold induction) at 12 h, and decreased thereafter. The *GhVHA-A* transcript level increased rapidly during the first 2 h of ABA treatment, increased markedly (by 4.9-fold) at 4 h, and decreased further at later time points. During low-temperature treatment, *GhVHA-A* expression increased to a peak (1.7-fold) at 2 h, and decreased thereafter.

Using qPCR, *GhVHA-A* was found to be differentially expressed in different cotton cultivars (**Figure 1C**). Indeed, expression was positively related to the level of drought tolerance determined for each cultivar (**Figure 1C**).

### *GhVHA-A* Silencing Compromised Dehydration Tolerance in Cotton

**FIGURE 2:**
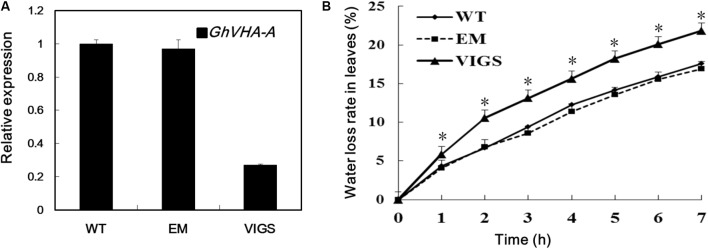
Dehydration tolerance analysis of *GhVHA-A* in cotton. **(A)** Gene expression of *GhVHA-A* in silenced and control plant leaves by quantitative real-time polymerase chain reaction (qRT-PCR) analysis. *GhUBQ7* was used as a control. Experiments were repeated three times with similar results. **(B)** Time course of water loss in detached leaves from WT, EM and VIGS plants, expressed as a percentage of the initial fresh weight at indicated intervals. Values were means ± SE from three biological replicates with 10 plants per replicate (^∗^*P* < 0.05; *t*-test).

To investigate the role of *GhVHA-A* in dehydration tolerance, we used a VIGS method to decrease expression of *GhVHA-A* in *G. hirsutum*. The VIGS, EM, and WT plants were subjected to PEG treatment and water deficit in a growth chamber. After 24 h PEG treatment, the WT and EM plants grew well. In contrast, the true leaves of VIGS plants showed severe wilting (**Supplementary Figure S2A**). Similar results were found in the water deficit experiments. After 15 days of water-withholding treatment, the wilting leaves of WT and EM plants was lower than that of the VIGS plants (**Supplementary Figure S2B**). Fourteen days after *Agrobacterium* infiltration, plants infiltrated with *GhCLA1* emerged with a photobleaching phenotype (**Supplementary Figure S2C**), show that VIGS-treated cotton plants were ready for subsequent analysis of gene loss of function. Silencing of *GhVHA-A* was further confirmed by RT-PCR analysis with RNA isolated from the leaves (**Figure 2A**). Furthermore, detached leaves of VIGS plants lost water more quickly than those of the WT and EM plants (**Figure 2B**).

### *GhVHA-A* Silencing Decreased the Tolerance of Cotton Under Dehydration Stress

**FIGURE 3:**
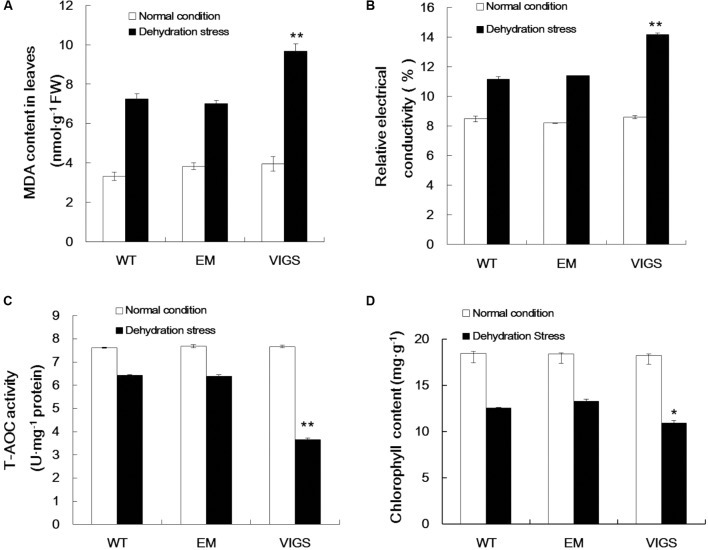
Changes in metabolites in *GhVHA-A*-silenced cotton under dehydration stress. **(A)** Malondialdehyde (MDA) content increased in *GhVHA-A*-silenced cotton under dehydration stress. **(B)** Relative electrical conductivity increased in *GhVHA-A*-silenced cotton under dehydration stress. **(C)** Activities of total antioxidative enzymes decreased in *GhVHA-A*-silenced cotton under dehydration stress. **(D)** Chlorophyll content decreased in *GhVHA-A*-silenced cotton under dehydration stress. All experimental data in leaves were tested after 15 days of water-deficit conditions. Values were means ± standard error (SE) of biological replicates (*n* = 5) (^∗^*P* < 0.05; ^∗∗^*P* < 0.01; *t*-test).

Dehydration stress conditions significantly enhanced MDA production in the leaves of VIGS plants, with a content of 9.68 nmol g^-1^ protein as compared with 7.02 nmol g^-1^ protein in EM plants (**Figure 3A**). Changes in plasma membrane permeability are a sensitive indicator of plant dehydration damage. The change in relative conductivity is an important index reflecting the size of electrolyte membrane permeability in plant tissue in stress conditions. As shown in **Figure 3B**, when exposed to dehydration stress, the relative conductivity was significantly increased in the leaves of VIGS plants (1.64-fold), with an average relative conductivity higher than EM and WT plants. Our analysis of MDA content and relative conductivity showed that the cell membrane systems of *GhVHA-A*-silenced cotton plants were seriously injured.

In addition, there is an important enzyme system in the plant body to protect against oxygen free radical damage. Consequently, we determined the activities of total antioxidant enzymes in the VIGS, EM, and WT plants. The activities of total antioxidant enzymes were significantly higher in EM and WT plants than in the VIGS plants under dehydration stress (**Figure 3C**). Compared with normal conditions, the chlorophyll content was 18.132 mg g^-1^, which decreased to 11.898 mg g^-1^ (34.4%) in the leaves of VIGS plants after 15 days of water-withholding treatment. This rate of decline is higher than that of EM and WT plants (**Figure 3D**). These results clearly indicated that silencing of *GhVHA-A* could decrease the resistance of cotton under dehydration stress.

### Overexpression of *GhVHA-A* Confers Dehydration Tolerance in Tobacco

**FIGURE 4:**
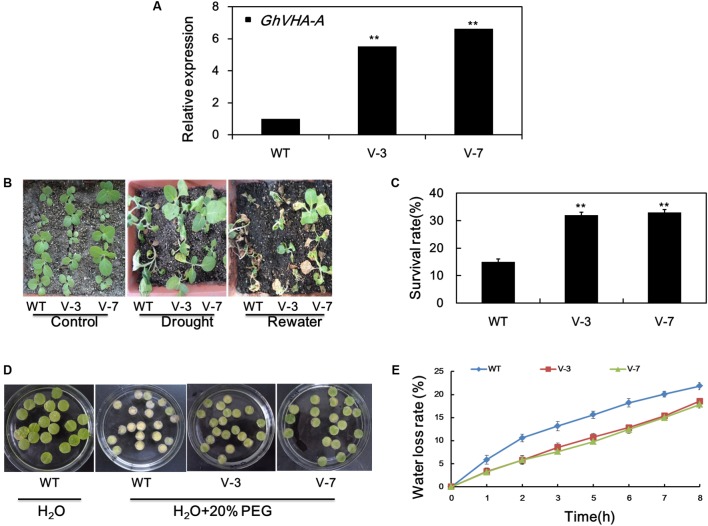
Overexpression of *GhVHA-A* increased the dehydration tolerance of transgenic tobacco plants. **(A)** Verification of *GhVHA-A* overexpression in transgenic tobacco lines by real-time reverse transcription (RT)-PCR. **(B)** Sensitivity of 4-week-old WT and transgenic plants to dehydration stress. Dehydration stress was imposed by withholding water for 15 days, and photographs were taken 7 days after re-watering. **(C)** Survival rate of the WT and two transgenic lines. Error bars represent SE of three independent experiments. **(D)** Leaf disks of WT and transgenic plants were soaked or not in distilled water containing 20% PEG 6000 for 7 days. **(E)** Water loss from detached leaves of WT and transgenic plants. Water loss was expressed as a percentage of initial fresh weight. Values are means of 10 leaves in each of three independent experiments.

To assess the *in vivo* function of *GhVHA-A*, transgenic tobacco plants overexpressing this gene were generated. The T_2_ generations of two homozygous transgenic lines, overexpression line 1 (V-3), and overexpression line 2 (V-7), were subjected to dehydration tolerance assays. The expression of *GhVHA-A* in the transgenic lines was elevated at least five-fold compared to WT plants (**Figure 4A**).

To further examine their dehydration-stress tolerance, water was withheld from 4-week-old seedlings of transgenic and WT plants for 15 days. The leaves of WT plants were wilted compared to those of transgenic plants (**Figure 4B**). After re-watering for 3 days, the survival rate of WT, transgenic line 1 (V-3), and transgenic line 2 (V-7) plants was 15, 33.3, and 35.5%, respectively (**Figure 4C**). Leaf disks from V-3 and V-7 plants stayed green after soaking in 20% PEG solution for 7 d, in contrast to the yellow leaf disks of the WT (**Figure 4D**). As shown in **Figure 4E**, the water-loss rate was higher in the WT than the V-3 plants following dehydration stress. Therefore, *GhVHA-A* overexpression could improve dehydration tolerance of tobacco plants.

Inhibition of root growth of T_2_ transgenic and WT plants under dehydration treatment was evaluated. Under normal growth conditions, the average root length of WT and transgenic tobacco plants did not differ significantly (**Supplementary Figure S3A,B**). However, the average root length of transgenic plants was higher than WT plants after dehydration stress (**Supplementary Figure S3C,D**). These results suggest that the deep root system of transgenic tobacco seedlings is involved in their improved tolerance to dehydration stress.

### Proline Content and Antioxidant Enzyme Activities Were Increased in Transgenic Tobacco Plants Under Dehydration Stress

**FIGURE 5:**
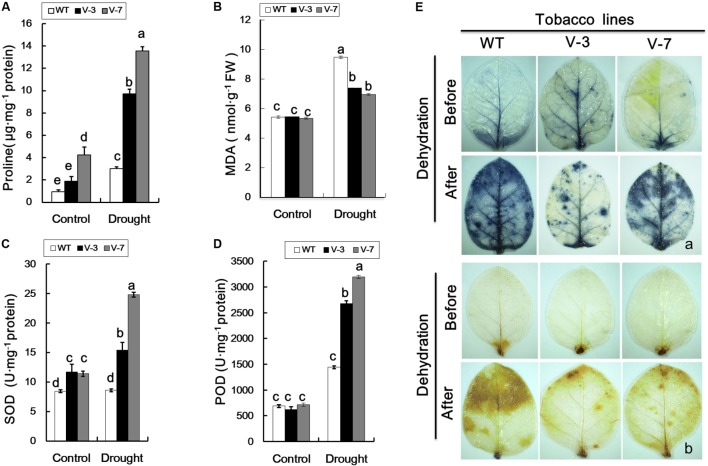
Overexpression of *GhVHA-A* reduced reactive oxygen species production and oxidative damage in transgenic plants under dehydration stress. **(A)** Proline contents of WT and transgenic plants under dehydration stress. **(B)** MDA contents of WT and transgenic tobacco plants under dehydration stress. **(C)** Superoxide dismutase (SOD) activity was increased in *GhVHA-A*-overexpressing tobacco plants under drought stress. **(D)** Peroxide dismutase (POD) activity of WT and transgenic plants. The data in **(A–D)** are means ± SE of three independent experiments. Different letters above columns indicate significant differences according to Duncan’s multiple range test (*P* < 0.05). **(E)** Representative photographs of staining with nitroblue tetrazolium (O_2_^-^, a) and 3,3′-diaminobenzidine (H_2_O_2_, b) in tobacco leaves before (upper panel) and after (lower panel) dehydration.

Compared with WT plants, the proline and MDA contents of V-3 and V-7 transgenic plants were not significantly different under normal growth conditions (**Figures 5A,B**). Following dehydration stress, the proline and MDA contents of both WT and transgenic plants were increased, however, the proline level in WT plants was significantly lower than in transgenic plants (**Figure 5A**). The SOD and POD activities of WT and transgenic plants were similar under normal growth conditions (**Figures 5C,D**). However, under dehydration stress, the SOD and POD activities of WT plants were significantly (*P* < 0.05) lower than those of transgenic plants (**Figures 5C,D**). These results were indicative of a regulatory role for *GhVHA-A* in the transgenic tobacco plant response to dehydration stress.

### ROS Accumulation Was Decreased in Transgenic Plants Exposed to Dehydration

Overexpression of *GhVHA-A* increased the proline content and SOD and POD activities, and decreased ROS accumulation, in transgenic tobacco plants under dehydration stress. Therefore, compared to the WT, the transgenic lines suffered from less serious membrane damage. Under normal growth conditions, very light histochemical staining of the leaves was observed with DAB and NBT, and the staining did not differ markedly between WT and transgenic lines (**Figure 5E**), implying a low level of ROS accumulation. However, exposure to dehydration result in extensive staining of the leaves of transgenic and WT plants, and WT plants exhibited deeper staining than the transgenic plants (**Figure 5E**). Therefore, ROS accumulation was greater in WT compared with transgenic plants.

### Enhanced Expression of Dehydration Stress-Responsive Genes in *GhVHA-A* Plants

**FIGURE 6:**
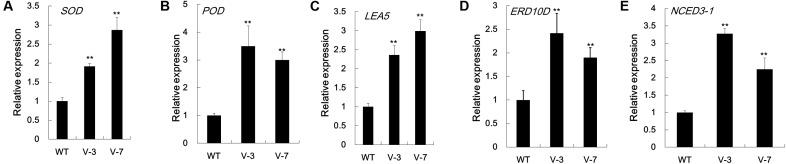
Expression analysis of stress-responsive genes in *GhVHA-A* transgenic tobacco. **(A)** Expression of antioxidant enzyme-coding gene *NtSOD* (AB093097). **(B)** Expression of antioxidant enzyme-coding gene *NtPOD* (ACN91229.1). **(C)** Expression analysis of stress-responsive gene *NtLEA5* in *GhVHA-A* transgenic tobacco. **(D)** Expression of the late embryogenesis abundant-coding gene *NtERD10D* (AB049338.1). **(E)** Expression of the abscisic acid biosynthesis gene *NtNCED3-1* (JX101472.1). RNA was extracted from leaf samples of WT and *GhVHA-A*-overexpressing plants (V-3 and V-7) after 7 days of withholding water, and reverse transcribed to synthesize complementary DNA. The transcript levels of stress-responsive genes were measured by qPCR. The *Nt18s* gene was used as an internal control. Data represent the means ± SE of three biological replicates (^∗^*P* < 0.05; *t*-test).

To increase our understanding of the molecular mechanism underlying the improved dehydration tolerance in *GhVHA-A*-overexpressing tobacco lines, the mRNA level of dehydration stress-related or stress-responsive genes was analyzed in transgenic lines and WT after 7 days of dehydration stress treatment. In agreement with the activities of antioxidant enzymes, the mRNA levels of antioxidant enzyme-coding genes, *NtSOD* and *NtPOD*, were significantly more in V-3 and V-7 than in WT plants under dehydration stress conditions (**Figures 6A,B**). qPCR analysis showed that the expression level of the stress defense protein-coding genes *NtLEA5* and *NtERD10D* increased in *GhVHA-A* transgenic lines under stress conditions (**Figures 6C,D**). The stress responsive target gene *NtNCED3-1* was upregulated almost 3.3- and 2.2-fold in V-3 and V-7 under dehydration conditions, respectively (**Figure 6E**). These results show that *GhVHA-A* can increase the expression of stress-defense genes under dehydration conditions, showing a novel role of *GhVHA-A* in the response to dehydration stress.

## Discussion

### *GhVHA-A* Was Induced by Abiotic Stress

*GhVHA-A* is differentially expressed in plants exposed to diverse abiotic stresses. Numerous examples are present in the literature of changes in mRNA, protein, or activity levels of V-ATPases in response to abiotic stress. For example, it was shown that the *VHA-A* transcript was increased in response to salt stress in wheat, tobacco, and sugar beet (Narasimhan et al., 1991; Lehr et al., 1999; Golldack and Dietz, 2001). In the present study, the expression of subunit A was induced by osmotic, salt, low-temperature, and ABA treatment stresses in *Gossypium hirsutum*.

Additionally, our qPCR analysis revealed that *GhVHA-A* was differentially expressed in different cotton cultivars, and the expression was significantly higher in drought-resistant than in drought-sensitive cultivars under dehydration stress. Therefore, the drought-resistant cultivars had higher VHA activities, likely due to enhanced *GhVHA-A* expression. This result may be directly related to the relationship between transcript, protein, and activity levels of V-ATPases.

### *GhVHA-A* Improves Plant Tolerance to Water Stress

Drought stress affects cotton yield, but the mechanism of drought resistance in cotton has not been revealed. Previous research found transgenic tobacco overexpression of apple *VHA-A* gene significantly increased dehydration resistance and salt tolerance (Dong et al., 2013, 2015). However, it is still not clear whether cotton VHA-A is involved in drought stress response. We explored whether transgenic tobacco plants overexpressing *GhVHA-A* showed improved tobacco performance under dehydration conditions. As expected, we found that transgenic tobacco plants had higher tolerance to dehydration compared with WT plants grown under dehydration conditions (**Figure 4B**). Previous studies have shown that decreased water loss contributes to dehydration tolerance in plants under dehydration stress (Yu et al., 2008). In our study, compared with WT plants, detached leaves of *GhVHA-A* transgenic lines had low water loss rate, indicating that it have stronger water holding capacity. In addition, previous studies have indicated that VHA is required for root growth of *Arabidopsis* under normal nutrient conditions and mild salt stress (Yamada et al., 2005). In this report, after dehydration stress, the average root length of transgenic plants was longer than WT plants. This suggested that VHA-A might be associated with root growth. Thus, overexpression of *GhVHA-A* in transgenic tobacco seedlings enhance dehydration tolerance, at least in part by retaining leaf moisture and promoting root growth.

Meanwhile, we found that silencing the *GhVHA-A* gene in *G. hirsutum* weakened its dehydration tolerance. Here, we used an *Agrobacterium*-mediated VIGS assay to silence the *GhVHA-A* gene to assess its effect on dehydration resistance of *G. hirsutum L*. We found that *GhVHA-A*-silenced cotton plants do not perform as well as WT and EM-treated cotton plants under dehydration conditions. Compared with WT and EM-treated seedlings, all physiological and biochemical indexes of *GhVHA-A*-silenced cotton seedlings changed greatly under dehydration stress (**Figure 3**). Based on our results, *GhVHA-A*-silenced cotton seedlings showed more severe wilting than the WT and EM-treated seedlings (**Supplementary Figure S2B**). Therefore, *GhVHA-A* plays an important role in plant optimal adjustment to water deficit conditions.

### *GhVHA-A* Improves Plant Tolerance to Oxidative Stress

A regulatory role for *VHA-A* in plants response to oxidative stress has been shown (Dong et al., 2013). In plants, drought also leads to ROS generation (Munné-Bosch and Peñuelas, 2003). Oxidative damage caused by the interaction of ROS with lipids, proteins, and DNA impairs the normal functions of cells. Therefore, ROS scavenging is a critical process for plants under stress. Proline is an important organic solute that accumulates in plants under drought stress, which is also considered to maintain the cellular redox status and protect membrane structure due to its properties as a scavenger of ROS. The increase in solute concentration results in enhanced osmotic adjustment (Ingram and Bartels, 1996; Zhang et al., 2014). In general, transgenic seedlings accumulate more proline than WT seedlings, indicative of enhanced osmotic adjustment (Dong et al., 2013). We report a similar finding in *GhVHA-A*-overexpressing tobacco seedlings. Thus, *GhVHA-A* may enhance the dehydration tolerance of transgenic tobacco seedlings by enhancing osmotic adjustment. Plants have a range of ROS-scavenging enzymes, including POD, SOD, and catalase. Among these, POD and SOD are key enzymes in the plant ROS scavenging system. In parallel, upregulation of ROS scavenging enzymes help providing plants with the best possible adaptations to water constraints (Randall and Sze, 1987). Previous studies have shown that *NtSOD* improves oxidative stress tolerance in tobacco (Slooten et al., 1995). In this study, the SOD and POD activities in *GhVHA-A*-overexpressing tobacco under dehydration stress were significantly higher than those in WT plants, and expression levels of the genes *SOD* and *POD* were significantly higher in *GhVHA-A*-overexpressing plants under dehydration stress (**Figures 6A,B**). In our study, we found that under dehydration stress conditions, transgenic seedlings suffered reduced cell damage, manifested as a decrease MDA content, which reflects the extent of lipid peroxidation in biomembranes indirectly and the level of membrane damage. Moreover, compared with EM plants, VIGS plants showed weak resistance to dehydration since MDA production was significantly enhanced under dehydration stress conditions (**Figure 3A**). MDA is one of the final products of lipid peroxidation in cell membrane. It is typically used as an index of lipid peroxidation, indicative of the degree of cell membrane lipid peroxidation and the ability of plants to withstand stress conditions. Thus, *GhVHA-A* improves dehydration stress tolerance in transgenic tobacco seedlings, at least in part by improving antioxidant enzyme activities and reducing oxygen-mediated damage.

Since the ROS level is associated with membrane integrity, histochemical staining with NBT and DAB was performed to evaluate the accumulation and localisation of ROS (O_2_^-^ and H_2_O_2_) in leaves subjected to dehydration stress (Shulaev and Oliver, 2006). Our results showed that under dehydration stress, the transgenic lines had a lower level of ROS compared with WT plants. Therefore, oxidative stress was weaker in transgenic plants compared to WT plants, which is in agreement with the enhanced stress tolerance of the former strain. The decreased level of ROS in the transgenic lines may have been due to their higher antioxidant enzyme activity (Kosová et al., 2015).

### *GhVHA-A* Influences the Expression of Stress-Related Genes

The *NtERD10D* genes encode group 2 late embryogenesis abundant (LEA) proteins, and the *NtLEA5* gene encodes a group 5 LEA protein. LEA protein association may be one mechanism safeguarding this group from irrevocable damage during quiescence in the dehydrated state (Chen et al., 2010). Both *NtERD10D* and *NtLEA5* function in binding water, stabilizing enzyme and macromolecular structures, and protecting cells from damage caused by abiotic stress (Becker et al., 1996; Hundertmark and Hincha, 2008; Liu et al., 2009). Under dehydration stress, the expression levels of stress-induced genes *NtERD10D* and *NtLEA5* were higher in *GhVHA-A* overexpressing plants (**Figures 6C,D**). The *NCED3* gene plays a key role in drought-stress inducible ABA biosynthesis (Seki et al., 2007). The high expression levels of the *NtNCED3-1* gene in *GhVHA-A*-overexpressing tobacco plants in dehydration stress (**Figure 6E**) showed that the overexpression of *GhVHA-A* may indirectly improve dehydration stress-induced ABA biosynthesis.

In summary, using an inverse genetics approach, we characterized the function of *GhVHA-A* gene in dehydration stress. Silencing of *GhVHA-A* in cotton plants led to an increase susceptibility to dehydration; whereas, overexpression of *GhVHA-A* in tobacco plants conferred lower water loss rate and higher average root length than WT plants, showing that these transgenic plants attained an improved tolerance to water limitation. Furthermore, *GhVHA-A* overexpression affected some stress-related physiological parameters (SOD activity and MDA level) in tobacco plants. These results suggest that VHA-A contributes to the plant tolerance to dehydration by enhancing osmotic adjustment (proline), reducing lipid peroxidation in biomembranes, and elevating SOD and POD activities. These results suggest that *GhVHA-A* plays an important role in response to drought stress in cotton, which helps us to understand the molecular mechanisms of drought resistance in cotton.

## Author Contributions

Y-yQ, GS, ZN, and NL designed the experiments and wrote the paper. NL performed all the experiments and analyzed the data. ZN helped to perform genetic transformation and partial data analysis. HZ, QC, WG, YC, ML, and ZN participated in the physiological assays and gene expression assays. All authors read and approved the manuscript.

## Conflict of Interest Statement

The authors declare that the research was conducted in the absence of any commercial or financial relationships that could be construed as a potential conflict of interest.

## Supplementary Material

FIGURE S1 V-ATPase was upregulated following dehydration stress. Data represent the means of two biological experiments, each with three replicates (a KK1543-0 h; b KK1543-24 h).

FIGURE S2 Dehydration response of *GhVHA-A*-silenced plantlets. **(A)** Phenotypes of *GhVHA-A*-silenced plantlets under polyethylene glycol (PEG) stress. WT, wild-type cotton plantlets; EM, cotton plantlets agroinfiltrated with the empty vectors pTRV1 and pTRV2; VIGS, cotton plantlets agroinfiltrated with the vectors pTRV2-*GhVHA-A* and pTRV1 to silence the *GhVHA-A* gene. **(B)** Phenotypes of *GhVHA-A*-silenced plantlets after 15 days of water-withholding treatment. **(C)** *CLA1*, cotton plantlets agroinfiltrated with the vectors pTRV2-*CLA1* and pTRV1 to silence the *CLA1* gene, used as a positive control with a phenotype of white colored leaves after silencing in cotton.

FIGURE S3 Root elongation in WT and transgenic tobacco plants exposed to dehydration stress. **(A)** Root elongation of WT and *GhVHA-A* transgenic plants under normal growth conditions. **(B)** Statistical analysis of the root length in **(A)**. **(C)** Root elongation of WT and transgenic tobacco plants exposed to dehydration stress for 7 days and then re-watered for 3 days. **(D)** Statistical analysis of the root length in **(C)**. All values represent means (±SE) of three replicates.
