# Supplementary figures and images for: The Gene Encoding Subunit A of the Vacuolar H+-ATPase From Cotton Plays an Important Role in Conferring Tolerance to Water Deficit
Source: Front Plant Sci. 2018 Jun 7;9:758. doi: 10.3389/fpls.2018.00758 (PMC6001365; doi:10.3389/fpls.2018.00758)

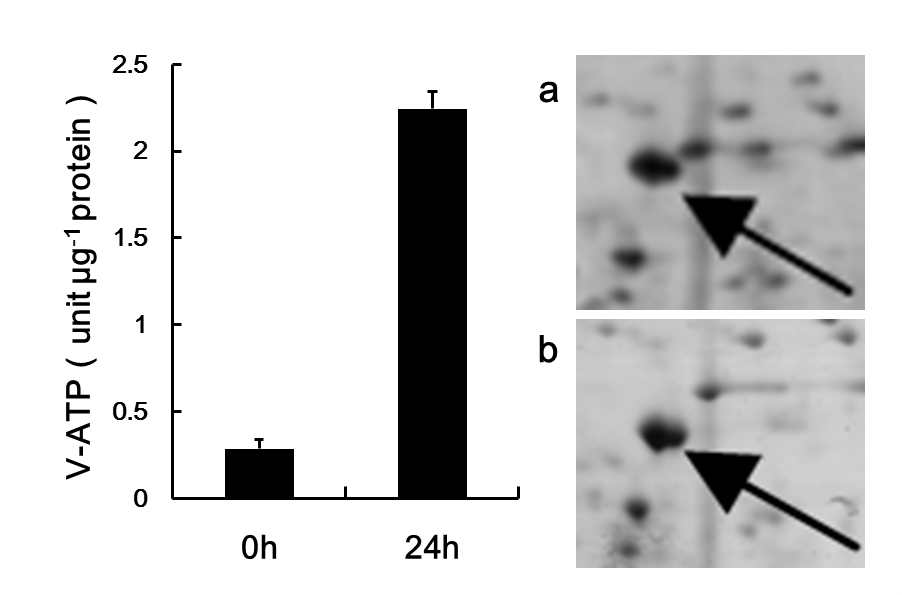

Supplement: FIGURE S1 — V-ATPase was upregulated following dehydration stress. Data represent the means of two biological experiments, each with three replicates (a KK1543-0 h; b KK1543-24 h). [file Image_1.TIF]

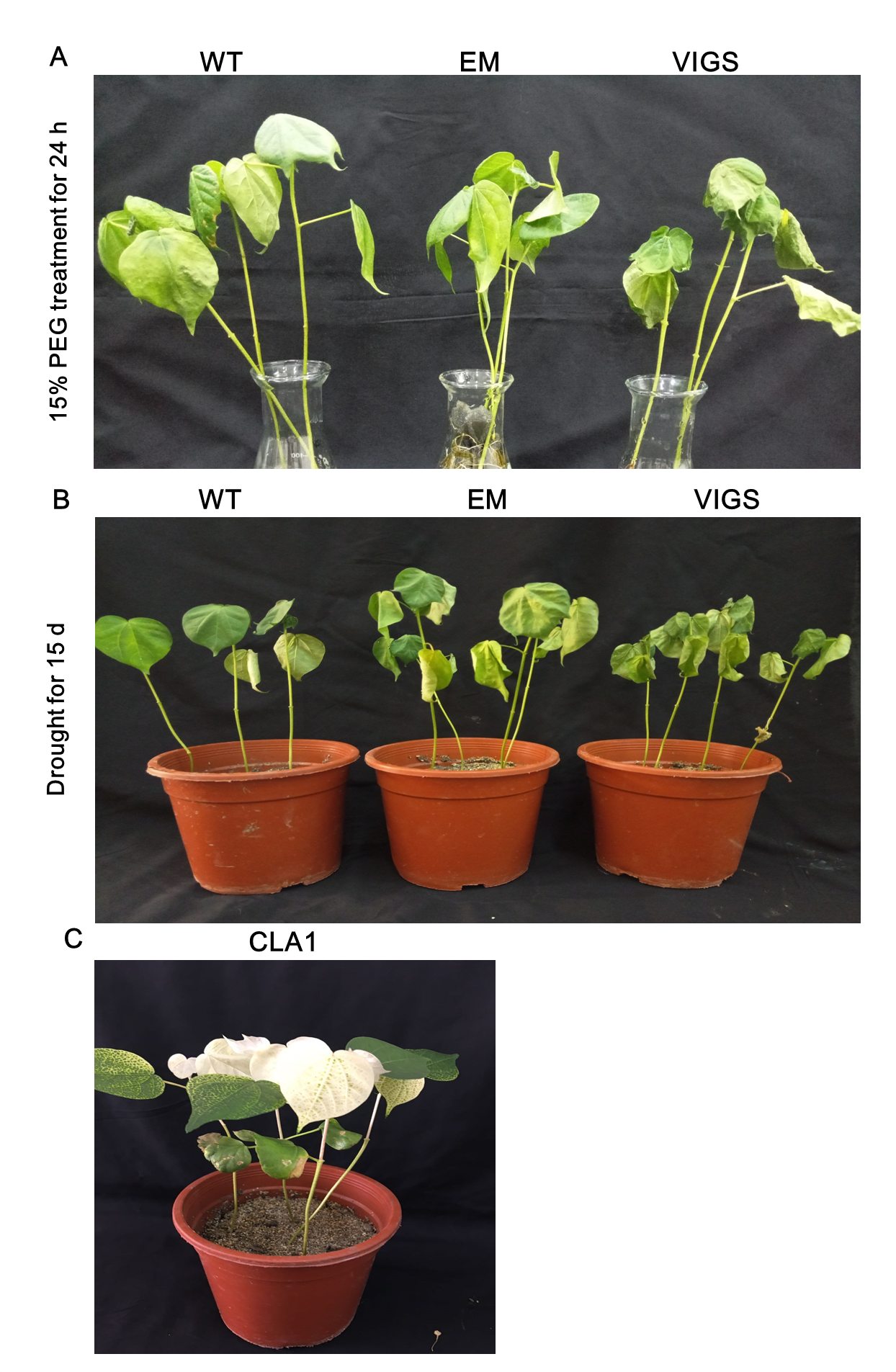

Supplement: FIGURE S2 — Dehydration response of GhVHA-A-silenced plantlets. (A) Phenotypes of GhVHA-A-silenced plantlets under polyethylene glycol (PEG) stress. WT, wild-type cotton plantlets; EM, cotton plantlets agroinfiltrated with the empty vectors pTRV1 and pTRV2; VIGS, cotton plantlets agroinfiltrated with the vectors pTRV2-GhVHA-A and pTRV1 to silence the GhVHA-A gene. (B) Phenotypes of GhVHA-A-silenced plantlets after 15 days of water-withholding treatment. (C) CLA1, cotton plantlets agroinfiltrated with the vectors pTRV2-CLA1 and pTRV1 to silence the CLA1 gene, used as a positive control with a phenotype of white colored leaves after silencing in cotton. [file Image_2.TIF]

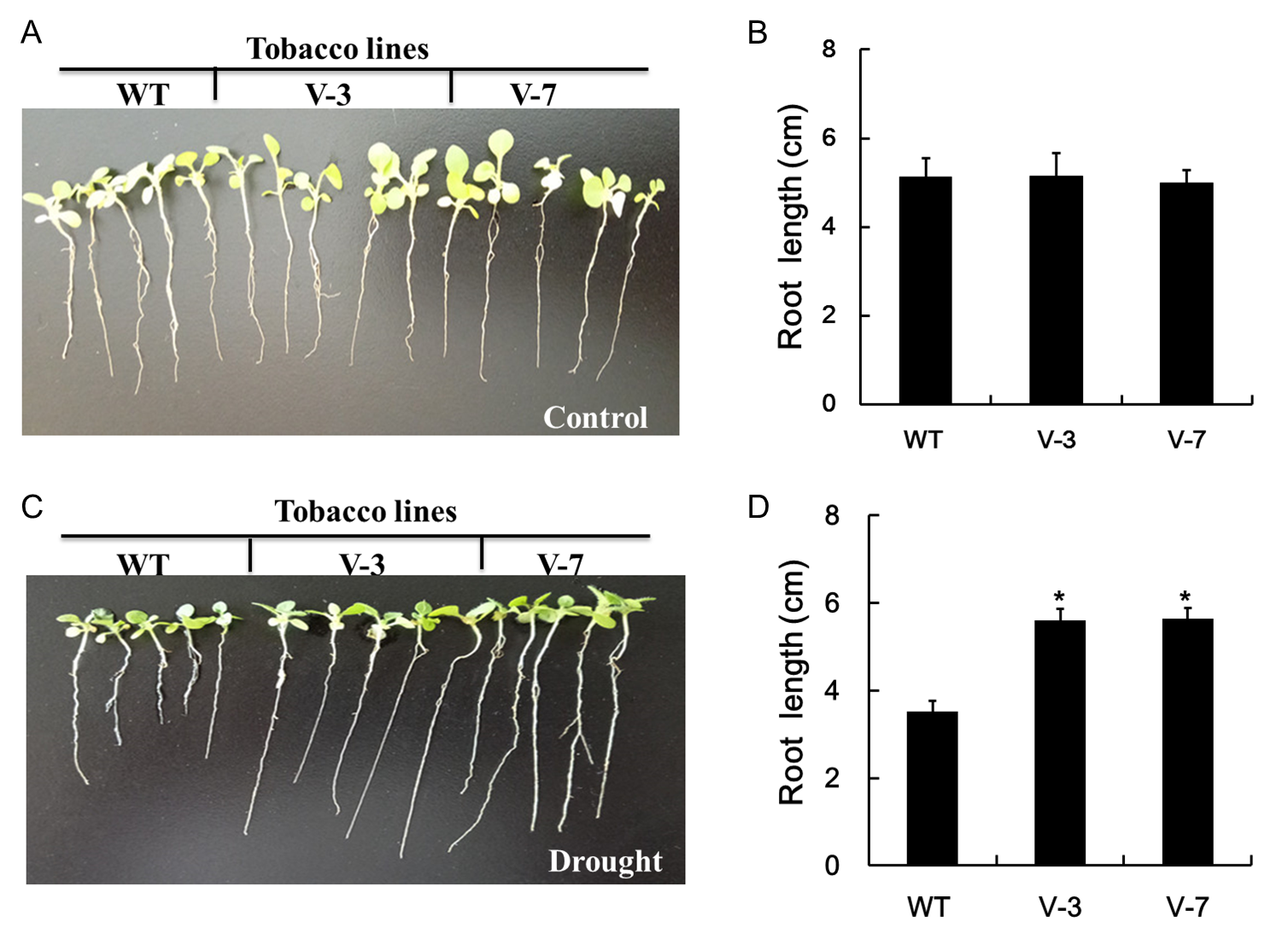

Supplement: FIGURE S3 — Root elongation in WT and transgenic tobacco plants exposed to dehydration stress. (A) Root elongation of WT and GhVHA-A transgenic plants under normal growth conditions. (B) Statistical analysis of the root length in (A). (C) Root elongation of WT and transgenic tobacco plants exposed to dehydration stress for 7 days and then re-watered for 3 days. (D) Statistical analysis of the root length in (C). All values represent means (±SE) of three replicates. [file Image_3.TIF]
